# Percutaneous Vertebral Augmentation with Polyethylene Mesh and Allograft Bone for Traumatic Thoracolumbar Fractures

**DOI:** 10.1155/2015/412607

**Published:** 2015-01-26

**Authors:** C. Schulz, U. Kunz, U. M. Mauer, R. Mathieu

**Affiliations:** Department of Neurosurgery, German Armed Forces Hospital Ulm, Oberer Eselsberg 40, 89081 Ulm, Germany

## Abstract

*Purpose*. In cases of traumatic thoracolumbar fractures, percutaneous vertebral augmentation can be used in addition to posterior stabilisation. The use of an augmentation technique with a bone-filled polyethylene mesh as a stand-alone treatment for traumatic vertebral fractures has not yet been investigated.* Methods*. In this retrospective study, 17 patients with acute type A3.1 fractures of the thoracic or lumbar spine underwent stand-alone augmentation with mesh and allograft bone and were followed up for one year using pain scales and sagittal endplate angles.* Results*. From before surgery to 12 months after surgery, pain and physical function improved significantly, as indicated by an improvement in the median VAS score and in the median pain and work scale scores. From before to immediately after surgery, all patients showed a significant improvement in mean mono- and bisegmental kyphoses. During the one-year period, there was a significant loss of correction.* Conclusions*. Based on this data a stand-alone approach with vertebral augmentation with polyethylene mesh and allograft bone is not a suitable therapy option for incomplete burst fractures for a young patient collective.

## 1. Introduction

It is still unclear whether operative or nonoperative treatment has a better clinical outcome in patients with incomplete thoracolumbar burst fractures (type A3.1 according to Magerl) without neurological deficit [1, 2]. In addition, there is also considerable controversy over what surgical strategy to use. Circumferential fusion is currently regarded as the standard surgical treatment, because posterior fixation alone is often associated with a loss of correction and hardware-related complications [3]. There is also controversy about the necessity of performing anterior fusion in addition to posterior instrumentation [4–7]. A combination of posterior stabilisation and transpedicular intravertebral cancellous bone grafting was not recommended in the past [8]. However, the use of percutaneous transpedicular kyphoplasty in addition to posterior instrumentation was regarded as an option for stabilising the anterior column especially in the management of osteoporotic fractures [9–11]. However, these osteoporotic fractures usually are the consequence of low energy accidents without intervertebral disc lesions and are not comparable to acute traumatic fractures of bone-healthy persons after high energy injuries from a biomechanical point of view. In a systematic review including 19 studies and a total of 727 cases, this approach was found to be superior to posterior stabilisation alone in terms of hardware failure and loss of correction [12]. Although the stand-alone use of percutaneous intravertebral augmentation using kyphoplasty also provides acceptable clinical results in the treatment of nonosteoporotic fractures, this approach appears to be inferior to a combination of posterior instrumentation and additional anterior augmentation in the management of acute traumatic thoracolumbar fractures especially in terms of their radiological results [13–17]. Another percutaneous vertebral augmentation (PVA) technique involves the use of an intravertebral mesh bag filled with allograft bone (spineoplasty [18]). A need for studies on the clinical course of patients undergoing PVA with mesh and allograft bone in the management of thoracic and lumbar fractures has already been identified [19]. No such studies, however, have been published so far.

## 2. Materials and Methods

From 2006 to 2008, 18 patients underwent stand-alone PVA with mesh and allograft bone (OptiMesh, Spineology) and only these cases were analysed in this retrospective study. Additionally 3 patients (showing a disruption of the posterior ligamentous complex) were treated by a combination of mesh bag PVA and bisegmental posterior stabilisation (these cases were not included into the further analyses of the stand-alone therapy). Diagnostic computerised tomography (CT) of the thoracic or lumbar spine was performed immediately after trauma in all cases. Additional magnetic resonance imaging scans were obtained in order to rule out an involvement of the posterior ligamentous complex and major traumatic disc lesions. The indication for the surgery in the presented analyses was a painful acute traumatic fracture (type A3.1 according to AO/Magerl) without sufficient response to conservative treatment. The case depending on individual decision for surgery was made by the responsible surgeon after consideration together with the patient. Percutaneous vertebral augmentation and reconstruction with an intravertebral mesh and morcelised bone graft were performed under general anaesthesia in prone position using a unilateral parapedicular approach as described by Chiu and Stechison [18]. For the correct placement of the working cannula a bidirectional fluoroscopy was used. After the unilateral left sided parapedicular insertion of a wire into the center of vertebral body the soft tissue was dilated using cannulas with increasing diameter (maximum 8 mm). Finally, a hollow cannula was inserted and the tip was positioned into the center of the fractured vertebral body. After correct placement of the hollow cannula, the mesh was inserted in center of the vertebral body followed by the piecemeal insertion of the allograft bone chips. Postoperative treatment consisted of analgesics, muscle relaxants, early functional mobilisation, and orthotic bracing in all cases.

In this retrospective study, only the data of 17 patients with an acute incomplete traumatic burst fracture of the thoracic or lumbar spine (between vertebral levels T6 and L3) without neurological deficit who were completely followed up over a period of one year after stand-alone PVA using a mesh filled with allograft bone were included (demographic data are shown in Table 1). One patient out of the initial cohort was lost to follow-up after 3 months and was excluded from the analyses. Further exclusion criteria were age >60 years, inadequate trauma severity, multiple vertebral fractures, disruption of the dorsal ligamentous complex revealed by MRI, any additional spine surgery to the stand-alone procedure, and known history of osteoporosis.

All procedures followed were in accordance with the ethical standards of the responsible committee on human experimentation (institutional and national) and with the Helsinki Declaration of 1975, as revised in 2008. The patients were informed that the data from their cases would be electronically saved and retrospectively analyzed, and they gave their written consent. Before and immediately after surgery (inpatient period) as well as 3, 6, and 12 months after surgery (outpatient period), outcomes were assessed using a visual analogue scale (VAS), the Denis pain and work scales [20], and the Macnab criteria [21]. The monosegmental and bisegmental sagittal endplate angles were measured (as described by Verheyden et al. [22]) using CT scans or lateral radiographs (CT scans were performed at the inpatient examinations. The outpatient examinations at 3 and 6 months were performed using CT scans or conventional radiographs. At the last outpatient examination 12 months after surgery a conventional spinal radiogram in standing position was performed in all patients). The fractures were scored according to the load-sharing classification (inaugurated by McCormack et al. [23]) using the initial CT scans. The orthotic braces were recommended to be worn for 12 weeks after surgery. After 6 weeks postoperatively a stepwise increasing of physical activity was allowed. After 12 weeks the patients were allowed to return to work without wearing braces.

Wilcoxon's test was used to detect significant differences in VAS scores and to compare monosegmental and bisegmental endplate angles at the different time points. The chi-squared test was used to analyse pain and work scale scores and the results for the Macnab criteria at the different time points. The level of significance was set at *P* < 0.05. Statistical analyses were performed using IBM SPSS Statistics 21.0.

## 3. Results

The median age of the participants was 34 years (range: 21–51). There were 6 female and 11 male patients. All cases were scored from minimum 3 to maximum 7 points according to the load sharing classification of McCormack et al. (for the individual data and results see Table 1). No case in the stand-alone treated cohort did show major disc lesions in the presurgical MRI. The median duration of surgery was 85 minutes (minimum: 55, maximum: 115, and standard deviation: 23.6). In no case did the intraoperative loss of blood exceed 100 mL. No intraoperative complications occurred.

Table 2 provides an overview of the pain and functional outcome scores and the results for the Macnab criteria. Immediately after surgery, all patients showed a significant improvement in pain and physical function. Twelve months after the procedure, pain and functional outcome scores were still significantly better than those obtained before surgery, as indicated by an improvement in the median VAS score (from 10 to 2; *P* < 0.001, Wilcoxon's test), the median pain scale score (from 5 to 2; *P* = 0.002, chi-squared test), and the median work scale score (from 5 to 2; *P* < 0.001, chi-squared test). At the time of discharge, all patients rated their surgical outcome as excellent or good (100% Macnab I or II). After 12 months, the surgical results were subjectively graded as excellent or good by only 13 of 17 patients (76.5% Macnab I or II). This decrease in patient satisfaction was significant (*P* = 0.043, chi-squared test).

**Figure 1:**
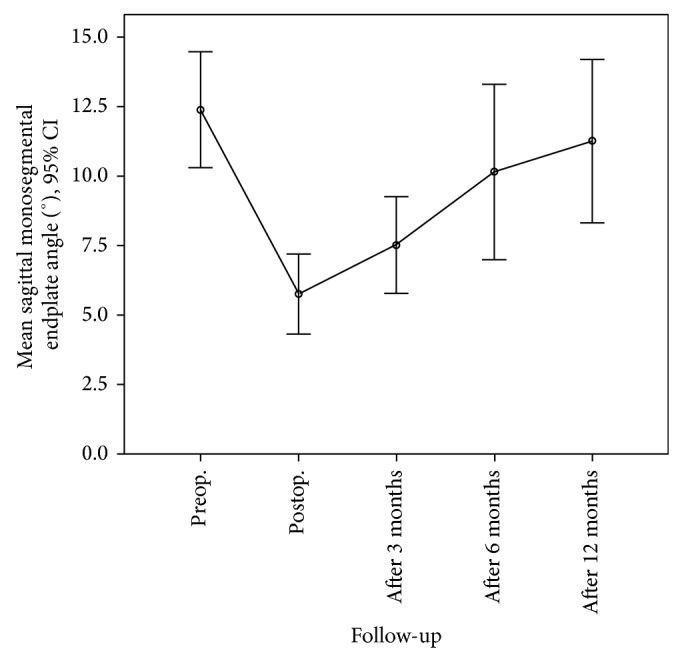
Mean monosegmental sagittal endplate angles from before surgery to 12 months after the procedure.

**Figure 2:**
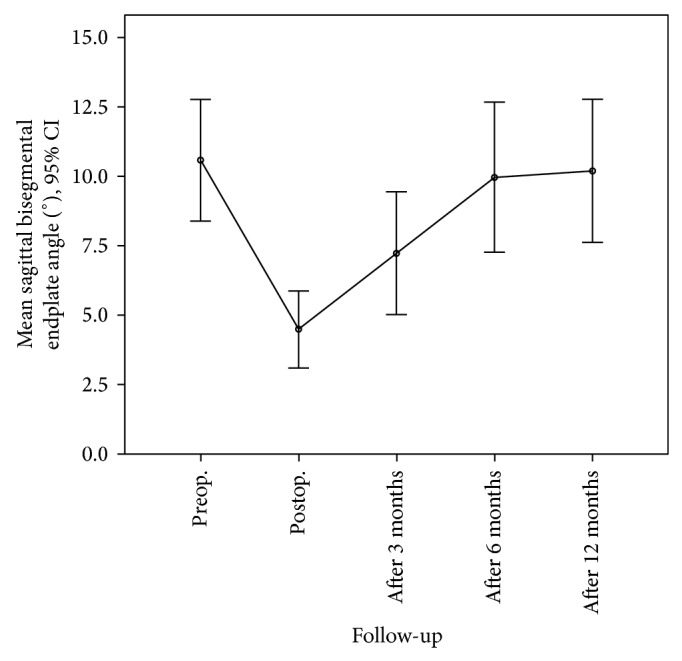
Mean bisegmental sagittal endplate angles from before surgery to 12 months after the procedure.

**Figure 3:**
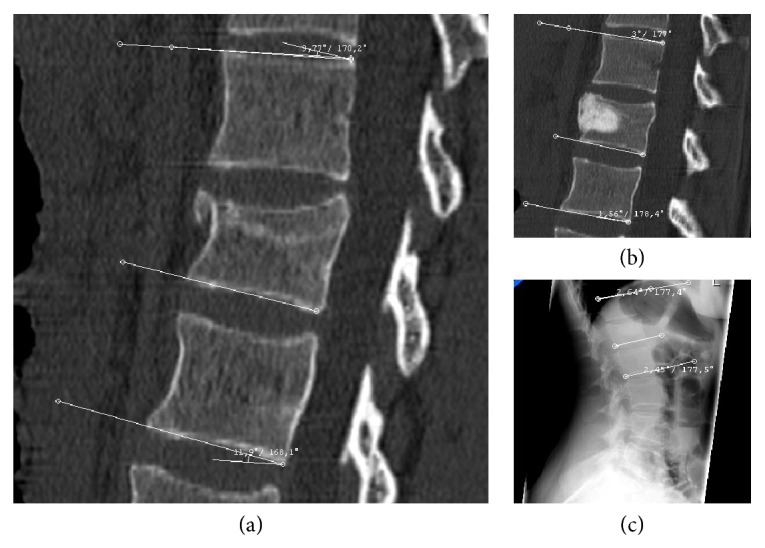
A twenty-nine-year-old woman sustained a fracture of L1 following a fall down stairs. Midsagittal reconstructed CT scans showed (a) kyphosis of 9.8° (monosegmental) and 11.9° (bisegmental) before surgery and (b) kyphosis of 3° (monosegmental) and 1.6° (bisegmental) immediately after stand-alone PVA with mesh and allograft bone. (c) Twelve months after surgery, the patient was completely symptom-free, had no neurological deficits, and was fully capable of performing her everyday activities. A lateral radiograph of the lumbar spine demonstrated kyphosis of 2.4° (monosegmental) and 2.5° (bisegmental).

**Figure 4:**
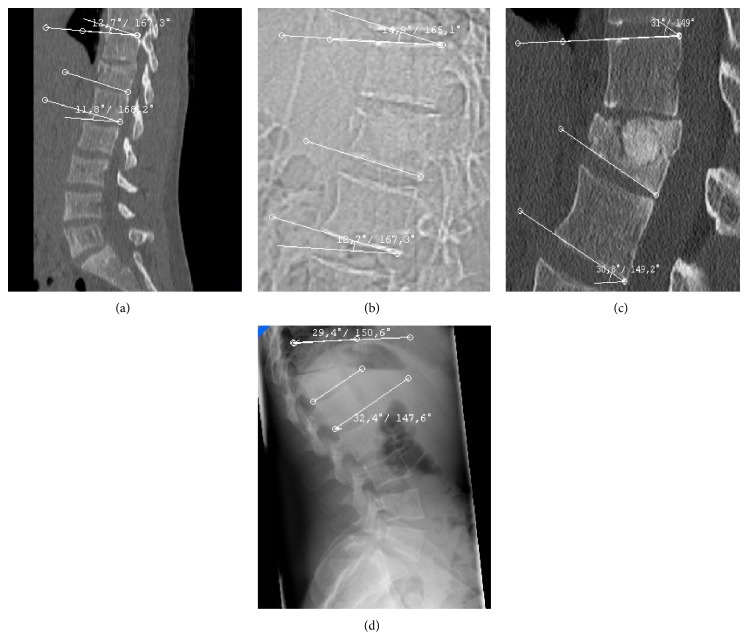
A twenty-one-year-old man sustained a fracture of T12 when he fell from a ladder. (a) A midsagittal reconstructed CT scan showed (a) kyphosis of 12.7° (monosegmental) and 11.8° (bisegmental). (b) A midsagittal reconstructed CT scan showed no significant correction immediately after surgery with kyphosis of 14.9° (monosegmental) and 12.7° (bisegmental). (c) Six months after surgery, the patient suffered from persistent back pain and a limited ability to perform physical activities. A midsagittal reconstructed CT scan showed kyphosis of 31° (monosegmental) and 30.8° (bisegmental). (c) Twelve months after surgery, the patient had no neurological deficits but occasionally required medications for back pain and was not fully capable of performing his everyday activities. (d) A lateral radiograph of the lumbar spine and thoracolumbar junction demonstrated kyphosis of 29.4° (monosegmental) and 32.4° (bisegmental). The patient did not wish to undergo surgical correction.

**Figure 5:**
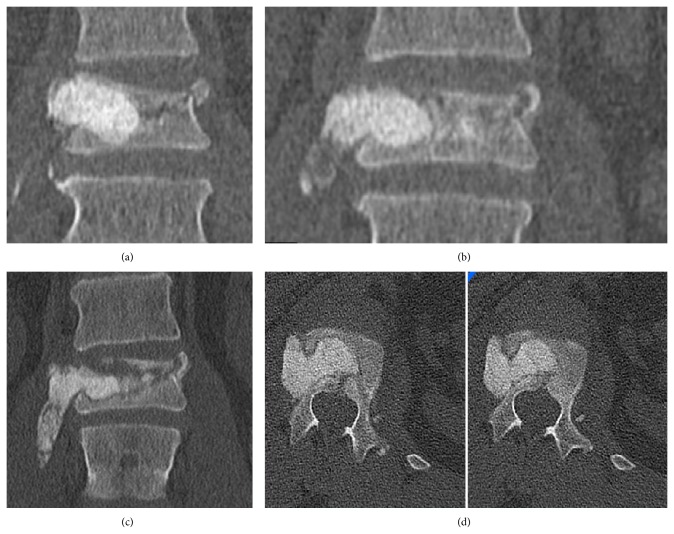
A forty-one-year-old man presented with a fracture of L1 after a fall. He underwent stand-alone PVA with mesh and allograft bone without complications. (a) A coronal reconstructed CT scan was obtained immediately after surgery and demonstrated the dislocation of the lateral border of the vertebral body on the right side. The mesh should have been placed more centrally. At that time point, the patient's symptoms had considerably improved compared to before surgery. The patient again suffered from back pain approximately one week after surgery when he began to perform more physically demanding activities. (b) A coronal reconstructed CT scan demonstrated progressive extravertebral displacement of the bone graft containment system. At this time point, the patient was almost free of symptoms, provided that he did not undertake strenuous physical activities. He did not wish to undergo surgery. Two weeks later, however, coronal (c) and axial (d) CT scans showed paravertebral extrusion of a major portion of the mesh and granular bone graft material. Surgical revision was then performed.

Figures 1 and 2 show how the mean monosegmental and bisegmental sagittal endplate angles changed during the twelve-month period. From immediately after trauma to the day after PVA patients showed a significant improvement in mean monosegmental kyphosis (from 12.4° to 5.8°, *P* < 0.001, Wilcoxon's test) and bisegmental kyphosis (from 10.6° to 4.5°, *P* < 0.001, Wilcoxon's test). During the following 12 months, there was a significant loss of correction (monosegmental: from 5.8° to 11.3°, *P* < 0.001, Wilcoxon's test; bisegmental: from 4.5° to 10.4°, *P* < 0.001, Wilcoxon's test). The difference between the degrees of kyphosis that were measured immediately after trauma and 12 months after surgery was no longer significant (monosegmental: 12.4° versus 11.3°, *P* = 0.166, Wilcoxon's test; bisegmental: 10.6° versus 10.2°, *P* = 0.45, Wilcoxon's test).  Figure 3 shows the best case and Figure 4 the worst case observed in this study. One patient was lost to follow-up. He required revision surgery probably because of inadequate closure of the polyethylene mesh with subsequent extravertebral leakage of granular bone graft material and resulting loss of correction (Figure 5).

## 4. Discussion

Compared with conservative treatment, stand-alone PVA using a mesh bag filled with allograft bone provides no major clinical and only minor radiological advantages [1, 2]. Compared with stand-alone kyphoplasty of acute traumatic thoracolumbar fractures, however, it yields comparable clinical and radiological results [12, 14, 15, 17]. However, stand-alone kyphoplasty is mainly used in osteoporotic patients, representing a different patient collective. Additionally, on a biomechanical aspect, kyphoplasty is an inconvenient strategy in incomplete burst fractures in patients with adequate trauma and unstable fracture situation. For this reason, it is not useful to compare stand-alone vertebral augmentation techniques between these two types of patients/fractures. The convincing results of stand-alone kyphoplasty in osteoporotic compression fractures cannot be estimated in or transferred to high energy acute traumatic fractures of the young adult with normal bone quality. During a twelve-month period, stand-alone PVA with mesh and allograft bone did not provide better pain and physical function scores than those reported in meta-analyses for posterior stabilisation alone or circumferential fusion [3–6]. This finding is supported by the results of a retrospective matched-cohort study in which we compared conservative treatment, circumferential fusion, and stand-alone PVA using a mesh filled with allograft bone (own unpublished data). There are a relevant number of patients after stand-alone treatment with vertebral augmentation who graded their outcome as insufficient (Macnab III or IV) and there is a significant and relevant postoperative loss of correction. An increasing number of authors attribute an important clinical role to the long-term restoration of sagittal alignment of the spine after the management of traumatic thoracolumbar fractures [24–27], although there are other authors who neither support nor refute this view [28] and others who do not share this opinion [29]. Against this background, surgeons should avoid surgical procedures that are likely associated with poor medium-term and long-term radiological results and that do not provide significant advantages compared to nonoperative treatment. This applies, for example, to stand-alone PVA with mesh and allograft bone, as indicated by the present study. Additionally the case of inadequate closure of the mesh (Figure 5), which may be a potential specific complication of the technique, is described for the first time in the literature reporting that PVA is not an incomplex technique. Although typical general surgical complications (comparable to vertebro- or kyphoplasty) were not detected during the 12-month period in the analysed 17 cases, this may just be a tribute to the low number of cases and not to the principled safety of this technique.

**Figure 6:**
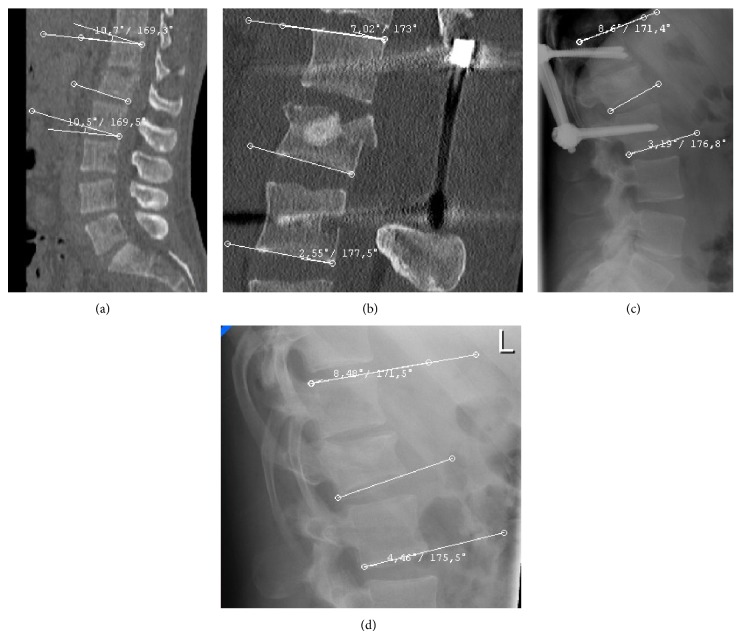
A twenty-two-year-old man sustained an L1 fracture in a motor vehicle accident. (a) Midsagittal reconstructed CT scans demonstrated initial kyphosis of 10.7° (monosegmental) and 10.5° (bisegmental). Since the patient showed a partial loss of motor function in the legs, he underwent laminectomy and posterior fixation. Three days later, PVA using a mesh bag filled with allograft bone was performed as an additional anterior procedure. (b) Following this procedure, a CT scan demonstrated kyphosis of 7° (monosegmental) and 2.6° (bisegmental). (c) After 12 months, there was no breakage of implants or loosening of screws. A lateral radiograph of the lumbar spine demonstrated kyphosis of 8.6° (monosegmental) and 3.2° (bisegmental). (d) Material was removed and the patient was reexamined after eighteen months. He had no neurological deficits and no back pain and was fully capable of performing his everyday activities. A lateral radiograph of the lumbar spine demonstrated kyphosis of 8.5° (monosegmental) and 4.5° (bisegmental).

Despite the fact that the loss of correction is less severe when PVA with mesh and allograft bone is combined with posterior stabilisation (Figure 6), the results of this treatment are not substantially different from those reported for other procedures combining percutaneous augmentation and posterior instrumentation [9–13, 15–17]. Likewise, a combination of PVA using a mesh filled with allograft bone and posterior stabilisation may be considered as an equal alternative to a combination of kyphoplasty or vertebroplasty with posterior stabilisation. After posterior stabilisation implant removal was reported to result in a loss of correction of −6.25° and residual kyphosis of 6.6° [30]. Therefore, a combination of PVA using a mesh bag filled with allograft bone and posterior stabilisation can at best be regarded as an alternative to posterior stabilisation alone and could be a suitable option in the management of traumatic thoracolumbar fractures.

Reported advantages of PVA with mesh and allograft bone include a more physiological modulus of elasticity and a higher potential for the biological incorporation of granular corticocancellous bone graft material [31] when compared to kyphoplasty or vertebroplasty using polymethyl methacrylate (PMMA) bone cement [32]. Twelve months after surgery, these postulated advantages did not play a relevant role in the group of patients presented here. PVA with mesh and allograft bone may have greater benefits when applied to osteoporotic vertebral compression fractures in a stand-alone manner [18].

The main limitations of this analysis are the use of a retrospective noncomparative observational approach, the small number of patients, and the short follow-up period. The high distribution of fracture levels (from Th 6 to L3) causes a tremendously different biomechanical behaviour of the fractures, determining different clinicoradiographic courses.

## 5. Conclusion

The use of stand-alone PVA using a mesh bag filled with allograft bone for the management of traumatic A3.1 fractures of the thoracic and lumbar spine results in a postoperative improvement of pain and physical activity scores. It is, however, also associated with a significant loss of correction. The benefits of PVA with mesh and allograft bone appear not to outweigh the complexity and potential complications of this technique as a stand-alone treatment. Based on this data a stand-alone approach with vertebral augmentation with polyethylene mesh and allograft bone is not a suitable therapy option for incomplete burst fractures for a young patient collective. Additionally there seems to be no benefit of this technique in comparison to conservative therapy.

## Figures and Tables

**Table 1:** Demographic data and individual clinicoradiographic results during 12-month follow-up.

Number	Age	Gender	Fracture level	Mechanism of injury	McCormack score	mSEA pre	bSEA pre	mSEA 12 m	bSEA 12 m	VAS 12 m	PAIN scale 12 m	WORK scale 12 m	MacNab 12 m
1^∗a^	29	F	L1	FGH	3	9.8	11.9	2.4	2.5	0	1	1	1
2	44	M	L2	MCA	4	10.3	7	7	4	0	2	3	3
3	46	M	TH12	MCA	4	10.6	8	5.8	5	2	2	2	1
4	25	M	TH11	BCA	5	15	14.5	13	14	1	3	4	3
5^∗b^	21	M	TH12	FGH	7	12.7	11.8	29.4	32.4	4	4	4	4
6	51	M	L1	CA	6	18	16	17	15	3	2	1	1
7	34	F	TH10	CA	5	16	14.5	18	16	3	3	4	3
8	43	F	L1	BCA	4	10	6	5.5	4	2	2	2	2
9	30	M	TH6	MCA	3	10	8	5.6	5	2	2	2	1
10	34	F	L2	MCA	5	15	14	13	12	3	2	3	2
11	32	M	L3	FGH	4	13.6	11.5	12	11	2	2	1	1
12	39	M	L1	MCA	3	11.5	8.5	8.2	7	0	1	1	1
13	36	F	TH12	CA	4	14	12	12	10.5	0	2	2	2
14	40	M	TH11	FGH	4	12.3	10.5	12.5	11.5	1	2	2	1
15	32	M	L1	MCA	3	11	8	8	8	0	1	2	2
16	38	M	TH12	CA	3	12	9.5	11.5	10	1	2	1	1
17	30	F	L2	FGH	4	11.8	8.5	10	9.5	1	2	2	1

mean						12.6	10.6	11.3	10.4				

mSEA: monosegmental sagittal endplate angle.

bSEA: bisegmental sagittal endplate angle.

CA: car accident.

MCA: motorcycle accident.

BCA: bicycle accident.

FGH: fall from great height.

VAS: Visual Analog Scale.

1^∗a^: case with the best clinicoradiographic results (see Figure 3).

5^∗b^: case with the worst clinicoradiographic results (see Figure 4).

**Table 2:** Clinical scores at follow-up.

	Before surgery	At discharge	Three months after surgery	Six months after surgery	12 months after surgery
**VAS** MED (MIN–MAX; SD)	10 (8–10; 0.7)	5 (3–7; 1.1)	4 (2–5; 1.0)	2 (0–5; 1.4)	2 (0–4; 1.3)

**Denis Pain Scale** (number of cases per grade)	I: 0, II: 0, III: 0, IV: 0, V: 17	I: 0, II: 0, III: 0, IV: 14, V: 3	I: 0, II: 0, III: 12, IV: 4, V: 1	I: 0, II: 4, III: 9, IV: 4, V: 0	I: 3, II: 11, III: 2, IV: 1, V: 0

**Denis Work Scale** (number of cases per grade)	I: 0, II: 0, III: 0, IV: 0, V: 17	I: 0, II: 0, III: 0, IV: 0, V: 17	I: 0, II: 0, III: 0, IV: 7, V: 10	I: 0, II: 8, III: 7, IV: 2, V: 0	I: 5, II: 7, III: 2, IV: 3, V: 0

**MacNab criteria** (number of cases per grade)	—	I: 8, II: 9, III: 0, IV: 0	I: 3, II: 14, III: 0, IV: 0	II: 5, II: 9, III: 3, IV: 0	I: 9, II: 4, III: 3, IV: 1

VAS: Visual Analogue Scale.

MED: median.

MAX: maximum.

MIN: minimum.

SD: standard deviation.

MacNab criteria I (excellent), II (good), III (fair), and IV (poor).
